# Postoperative sensorineural hearing loss associated with prolonged hypocalcemia

**DOI:** 10.1210/jcemcr/luag298

**Published:** 2026-10-05

**Authors:** Samantha Esposito, Allen T Yu, Laura Barthold, Denise Lee

**Affiliations:** Department of Surgery, Icahn School of Medicine at Mount Sinai, New York, NY 10029, USA; Department of Surgery, Icahn School of Medicine at Mount Sinai, New York, NY 10029, USA; Department of Surgery, Icahn School of Medicine at Mount Sinai, New York, NY 10029, USA; Department of Surgery, Icahn School of Medicine at Mount Sinai, New York, NY 10029, USA

**Keywords:** parathyroidectomy, sensorineural hearing loss, hypocalcemia

## Abstract

A 57-year-old woman with end-stage renal disease and secondary hyperparathyroidism underwent subtotal parathyroidectomy and developed profound postoperative hypocalcemia consistent with hungry bone syndrome, followed by acute sensorineural hearing loss on postoperative day 3. Calcium plays a critical role in cochlear hair cell transduction and auditory signaling, and disruptions in calcium homeostasis have been associated with hearing loss, although primarily in chronic or syndromic conditions rather than acute postoperative states. This case underscores the importance of aggressive calcium repletion in hungry bone syndrome, as well as early recognition of atypical symptoms such as hearing changes to enable prompt multidisciplinary management and optimize patient outcomes.

## Introduction

Secondary hyperparathyroidism is a common complication of end-stage renal disease (ESRD), leading to electrolyte disruptions, bone demineralization with associated pain, and fatigue [1]. Initial medical management with vitamin D supplements, phosphate binders, and calcimimetics are trialed; however, if symptoms persist, parathyroidectomy can be considered. Hungry bone syndrome (HBS) frequently occurs after parathyroidectomy, characterized by prolonged and profound hypocalcemia lasting more than four days postoperatively [2-4]. Although HBS typically presents with muscle spasms, paresthesia, and fatigue, this case reports on a patient who developed sensorineural hearing loss after prolonged hypocalcemia following a parathyroidectomy.

## Case presentation

This case presents a 57-year-old female patient with a history of hypertension, atrial fibrillation on apixaban, prior non-ST elevation myocardial infarction, hyperlipidemia, chronic obstructive pulmonary disease, type 2 diabetes mellitus on insulin, osteopenia, ESRD on hemodialysis for the past 12 years, and secondary hyperparathyroidism. She was initially evaluated for parathyroidectomy in 2023; however, surgery at that time was deferred because of improvement in alkaline phosphatase to 160 U/L (SI: 2.667 μkat/L; reference range 38-126 U/L [SI: 0.73-2.45 μkat/L]) from 468 U/L (SI: 7.802 μkat/L), and PTH of 431 pg/mL (SI: 43.328 pmol/L; reference range: 10-65 pg/mL [SI: 1.0-7.0 pmol/L]) and lack of symptoms. She was started on cinacalcet 120 mg 3×/week at that time. She was reevaluated 2 years later after her PTH increased to 995 pg/mL (SI: 100.027 pmol/L) and she became severely symptomatic with fatigue and debilitating bone and joint pain. Given that her multidisciplinary care team concluded the most likely etiology was her secondary hyperparathyroidism, after thorough discussion, parathyroidectomy was recommended. Of note, she was unfamiliar with cinacalcet and could not report if she had been taking it, despite reports that it had been obtained from her pharmacy. Prior to surgery, she completed a magnetic resonance imaging scan that was unable to demonstrate a parathyroid adenoma; a thyroid ultrasound demonstrated a subcentimeter nodule (0.7 cm × 0.4 cm × 0.6 cm) in the upper posterior aspect of the left thyroid.

Intraoperatively, a neck ultrasound demonstrated enlarged nodules at the right superior, right inferior, and left inferior aspects of the thyroid, likely representing parathyroid adenomas. The right superior, left superior, and left inferior parathyroid glands were fully excised, and about two-thirds of the right inferior parathyroid gland was excised, leaving a 1-cm remnant. Intraoperative PTH levels were monitored: 0 minutes - 102 pg/mL (SI: 10.254 pmol/L), 5 minutes - 92 pg/mL (SI: 9.249 pmol/L), 10 minutes - 82 pg/mL (SI: 8.243 pmol/L), 15 minutes - 80 pg/mL (SI: 8.042 pmol/L)), 20 minutes - 77 pg/mL (SI: 7.741 pmol/L), and 25 minutes - 75 pg/mL (SI: 7.540 pmol/L). Frozen biopsies of each sample were confirmed as parathyroid adenomas. The right forearm was prepped and ∼30 to 40 mg of macerated parathyroid tissue were separated into 4 pockets within the brachioradialis muscle. The patient tolerated the procedure well and was brought to the postanesthesia care unit in stable condition and transferred to the floor for further monitoring.

## Diagnostic assessment

The patient initially recovered as expected with minimal throat pain, tolerating diet and without numbness/tingling periorally or in her digits. Her calcium decreased to 6.0 mg/dL (SI: 1.5 mmol/L; reference range: 8.5-10.5 mg/dL [SI: 2.1 to 2.6 mmol/L]) on postoperative day 1 (POD) (PTH 50 pg/mL (SI: 5.027 pmol/L)), remained 6.4 mg/dL (SI: 1.6 mmol/L) (PTH 53 pg/mL (SI: 5.329 pmol/L)), and 6.6 mg/dL (SI: 1.65 mmol/L) (PTH 58 pg/mL (SI: 5.831 pmol/L)) on POD2 and POD3, respectively. On POD 3, the patient reported decreased hearing in her right ear with normal hearing in her left. She denied otalgia or otorrhea but endorsed tinnitus. Her calcium and calcitriol dosing was increased and she attended hemodialysis with a calcium bath. On POD4, otolaryngology (ENT) was consulted and an audiogram was completed that demonstrated thresholds ranging from 65 dB at 250 Hz worsening to 95 to 100 dB at 2000 to 4000 Hz representing severe to profound hearing loss in the right ear. The left ear thresholds ranged from 45 dB at 250 Hz to 35 to 40 dB at 1000 to 2000 Hz, and worsening to 40 to 45 dB at 4000 to 8000 representing mild-moderate hearing loss. A high-dose steroid taper (prednisone 60 mg × 7 days followed by taper) was started on POD5. On POD6, she reported slight improvement in her hearing.

## Treatment

Her calcium and PTH were monitored throughout her hospitalization; calcium carbonate and calcitriol were adjusted accordingly with additional intravenous (IV) calcium gluconate and calcium baths during hemodialysis (HD) sessions as needed (Table 1, Table 2, and Fig. 1). She was discharged on POD7 on calcium carbonate 1250 mg 4 times per day, calcitriol 0.5 mcg 4 times per day, and outpatient follow-up with endocrine surgery for further medication management and ENT for repeat audiogram and magnetic resonance imaging of the internal auditory canal.

**Table 1:** Calcium treatment during postoperative course

Treatment	POD0	POD1	POD2	POD3	POD4	POD5	POD6	POD7
Calcium carbonate (mg)	2500 loading dose	1250 q6h	2500 q6h	2500 q4h	2500 q4h	2500 q6h	2500 q6h	2500 q6h
Calcitriol (mcg)	3 loading dose	1 q6h	1 q6h	1 q4h	1 q4h	1 q6h	0.5 q6h	0.5 q6h
IV calcium gluconate (g)	0	3	4	4	0	0	0	0
HD calcium Bath (mEq/L)	No HD	No calcium bath at HD	No HD	3	No HD	3.5	No HD	2.5

Abbreviations: HD, hemodialysis; POD, postoperative day; q4h, every 4 hours; q6h, every 6 hours.

**Table 2:** Postoperative calcium, ionized calcium, and PTH monitoring throughout hospital stay

Time from surgery	−24:00:00	0:00:00	5:00:00	14:00:00	26:00:00	32:00:00	37:00:00	45:00:00	52:00:00	57:00:00
Calcium (mg/dL, reference range: 8.5-10.5 mg/dL; SI: mmol/L, reference range: 2.1-2.6 mmol/L)	8.2 mg/dL (SI: 2.02 mmol/L)	7.5 mg/dL (SI: 1.87 mmol/L)	6.6 mg/dL (SI: 1.65 mmol/L)	6 mg/dL (SI: 1.5 mmol/L)	6.8 mg/dL (SI: 1.7 mmol/L)	6.6 mg/dL (SI: 1.65 mmol/L)	6.5 mg/dL (SI: 1.62 mmol/L)	6.4 mg/dL (SI: 1.6 mmol/L)	6.4 mg/dL (SI: 1.6 mmol/L)	6.2 mg/dL (SI: 1.55 mmol/L)
Ionized calcium (mmol/L, reference range: 1.14-1.29 mmol/L; SI: units and range are the same)	0.99 mmol/L	0.82 mmol/L								
PTH (pg/mL; reference range: 10-65 pg/mL; SI: pmol/L, reference range 1.0-7.0 pmol/L)	372 pg/mL (SI: 39.45 pmol/L)	55 pg/mL (SI: 5.83 pmol/L)		50 pg/mL (SI: 5.3 pmol/L)			53 pg/mL (SI: 5.62 pmol/L)			58 pg/mL (SI: 6.15 pmol/L)

Time from Surgery	63:00:00	81:00:00	84:00:00	96:00:00	108:00:00	120:00:00	132:00:00	144:00:00	156:00:00
Calcium (mg/dL, reference range: 8.5-10.5 mg/dL; SI: mmol/L, reference range: 2.1-2.6 mmol/L)	6.6 mg/dL (SI: 1.65 mmol/L)	7.5 mg/dL (SI: 1.87 mmol/L)	7.7 mg/dL (SI: 1.92 mmol/L)	7.2 mg/dL (SI: 1.8 mmol/L)	7.7 mg/dL (SI: 1.92 mmol/L)	10.2 mg/dL (SI: 2.54 mmol/L)	9.9 mg/dL (SI: 2.47 mmol/L)	9.7 mg/dL (SI: 2.42 mmol/L)	9.2 mg/dL (SI: 2.3 mmol/L)
Ionized Calcium (mmol/L, reference range: 1.14-1.29 mmol/L; SI: units and range are the same)		0.92 mmol/L	0.97 mmol/L	0.91 mmol/L	0.99 mmol/L	1.25 mmol/L	1.24 mmol/L	1.24 mmol/L	1.14 mmol/L
PTH (pg/mL; reference range: 10-65 pg/mL; SI: pmol/L, reference range 1.0-7.0 pmol/L)			70 pg/mL (SI: 7.42 pmol/L)		88 pg/mL (SI: 9.33 pmol/L)		26 pg/mL (SI: 2.76 pmol/L)		32 pg/mL (SI: 3.39 pmol/L)

## Outcome and follow-up

During her 1-week follow-up appointment after completing the steroid taper, she reported continued tinnitus and fullness of her right ear without otalgia, otorrhea, or dizziness/vertigo. Her repeat audiogram resulted in thresholds ranging from approximately 90 dB at 250 Hz to 70 to 80 dB across mid and high frequencies in the right ear, depicting persistent severe sensorineural hearing loss. There were mild improvements in the left ear hearing with thresholds of 50 dB at 125 to 250 Hz, improving to ∼25 to 35 dB at 1000 to 3000 Hz, then worsening slightly to ∼30 dB at higher frequencies. She received a right intratympanic steroid injection (dexamethasone 0.5 mL) with plans for a second intratympanic injection in 1 week. Audiology evaluation after the second intratympanic injection was not available.

## Discussion

Secondary hyperparathyroidism in patients with ESRD is the result of the kidneys’ decreased ability to excrete phosphorus and convert vitamin D into its physiologically active form, 1,25-cholecalciferol (calcitriol), leading to decreased uptake of calcium in the gastrointestinal tract [1]. This triggers a persistent feedback response causing increased secretion of PTH, typically at levels >150 pg/mL, in efforts to increase the amount of calcium in circulation [1, 2]. With elevated levels of PTH and calcium, complications such as bone mineral disorders and vascular and soft-tissue calcification can occur [2]. Symptoms such as weakness, fatigue, and bone pain, as seen in the current patient, are common and are initially treated with vitamin D analogues and calcimimetics [2]. When medications do not adequately control a patient's symptoms, surgical intervention can be considered. Long-term data suggest that approximately 15% of patients require parathyroidectomy 10 years after a diagnosis of ESRD, increasing to nearly 38% after 20 years [2]. The current patient follows this pattern as she required parathyroidectomy 12 years following ESRD diagnosis.

HBS is a common complication of parathyroidectomy, with an incidence as high as 82% in patients on HD [5]. It is defined as a prolonged and profound decrease in serum calcium (<8.4 mg/dL) or hypocalcemia lasting more than 4 days following surgery [2, 4]. When the parathyroid tissue is removed, there is a sudden drop in PTH levels causing unopposed osteoblast activity leading to a rapid uptake of calcium into previously demineralized bone [2-4]. Clinically, this presents as severe hypocalcemia leading to neuromuscular symptoms, such as perioral numbness, extremity tingling, carpopedal spasms, and positive Chvostek and Trousseau signs [4]. Risk factors for HBS include elevated alkaline phosphatase, blood urea nitrogen and PTH, radiological evidence of bone disease, higher body mass index, and in cases of secondary hyperparathyroidism, younger age, and lower preoperative calcium [6]. Management focuses on aggressive calcium repletion with intravenous calcium, along with adjustments in oral calcium and calcitriol supplementation until appropriate calcium levels are maintained. In HD patients, the use of high-calcium dialysate can also be used to raise serum calcium levels [2-4]. Additionally, before parathyroidectomy, patients can be started on calcium and calcitriol supplementation to further prevent HBS from occurring, which may have helped avoid this present hearing loss for the current patient.

Although the current patient did not exhibit the traditional symptoms of acute hypocalcemia, it is possible that the sudden shift in calcium homeostasis triggered her sensorineural hearing loss (SNHL) on POD3. SNHL is defined as an impairment in sound perception or neural signal conduction due to damage to the auditory pathways, including the cochlear hair cells and auditory nerve [7]. Typical auditory transduction is the result of vibrations of the basilar membrane deflecting hair cells causing ion channels to open, allowing for calcium influx, depolarization of the cells, and neurotransmitter release [8]. Disruptions in calcium concentrations within the endolymph (maintained at 20 μM) and perilymph (5 mM), or impaired calcium signaling, can result in hearing loss [8]. Correlations between hearing loss and hypocalcemia have only been noted in a chronic setting, particularly in patients with the autosomal dominant disorder hypoparathyroidism, deafness, and renal dysplasia (HDR) syndrome, caused by mutations in the GATA3 gene [9]. In the 118 cases of HDR, patients most often present with bilateral SNHL as an early manifestation, with hypocalcemia presenting later [9]. The management of an HDR patient's hypocalcemia and SNHL is particularly complex, as standard therapies such as calcium, calcitriol, or recombinant PTH have not been used due to concerns regarding underlying renal dysplasia and the need for careful long-term monitoring [9].

The current patient developed acute SNHL in the immediate postoperative period following parathyroidectomy. Preoperatively, she exhibited several risk factors for the development of HBS, including elevated alkaline phosphatase, radiographic evidence of osteopenia, and increased body mass index, all of which likely contributed to her postoperative course. However, SNHL associated with prolonged hypocalcemia following parathyroidectomy has not been previously reported. To date, most described associations between hypocalcemia and hearing loss are either chronic or syndromic and are typically present bilaterally. The timing of symptom onset in this patient (POD3) corresponds with the expected onset of HBS. Given the well-established role of calcium in cochlear hair cell function, it is plausible that acute hypocalcemia contributed to impaired auditory signal transduction, resulting in SNHL, although definitive causation cannot be concluded.

Management of postoperative hypocalcemia in this patient required aggressive calcium and calcitriol supplementation. Throughout her hospitalization, she received high-dose oral (PO) calcium and calcitriol with IV calcium gluconate and calcium baths during HD. She was ultimately discharged on calcium carbonate 1250 mg 4 times daily and calcitriol 0.5 mcg 4 times daily. These interventions align with established treatment strategies for HBS, which emphasize rapid and sustained correction of calcium deficits. However, despite the aggressive supplementation, the patient still developed acute SNHL. Mainstay treatment of acute SNHL is corticosteroids because of their anti-inflammatory effects and potential to reduce cochlear injury [6]. The patient's reported improvement in hearing following initiation of steroids and calcium repletion suggests that early recognition and additional management with glucocorticoids may be beneficial.

This case highlights a rare but clinically significant potential complication of parathyroidectomy in patients with secondary hyperparathyroidism. Although the relationship between calcium dysregulation and hearing loss is well established at a mechanistic level, clinical correlations remain limited. This underscores the importance of close postoperative monitoring of calcium levels, early recognition of atypical symptoms such as hearing changes, and prompt multidisciplinary management to optimize outcomes.

**Figure 1:**
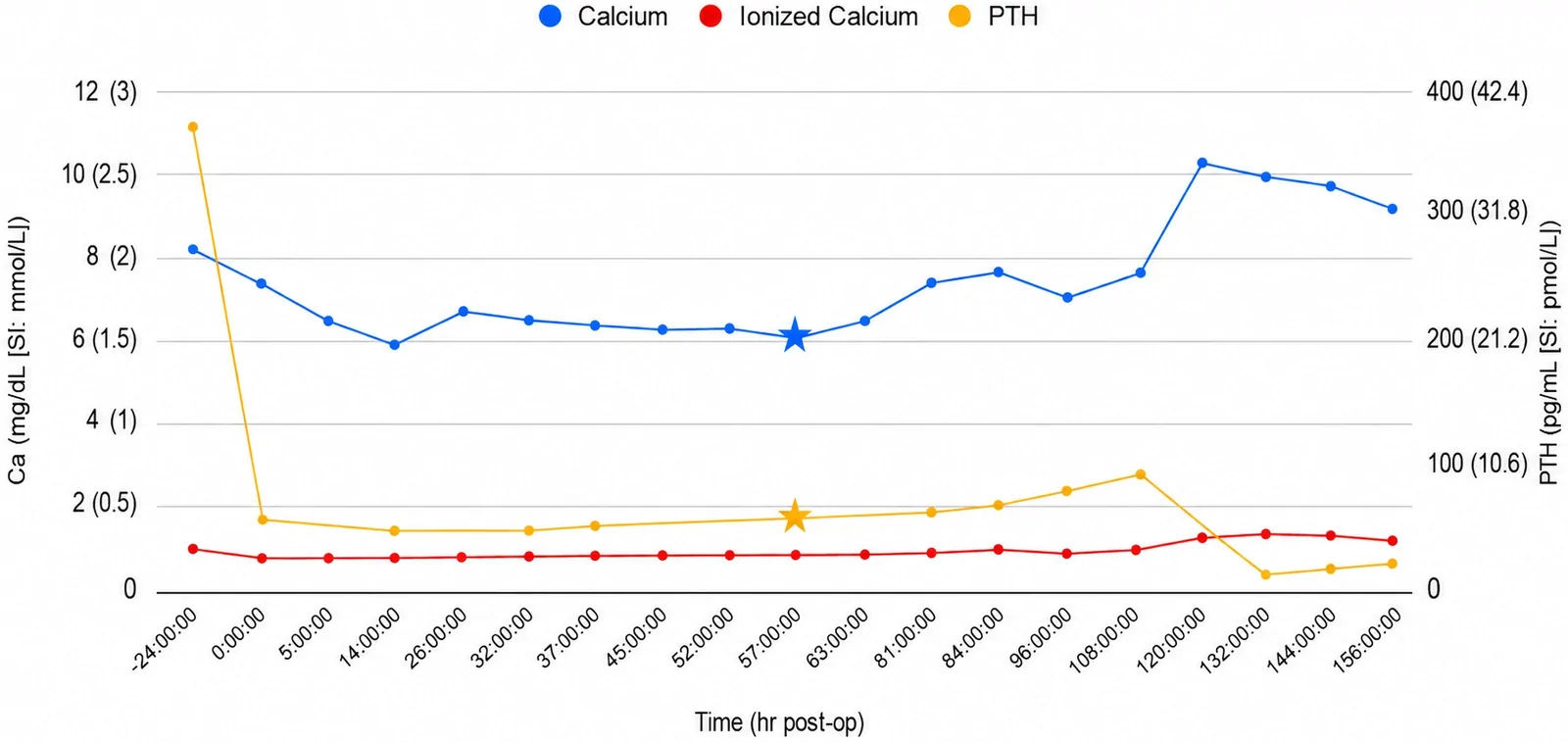
Left vertical axis demonstrates discrete data for calcium (mg/dL, reference range: 8.5-10.5 mg/dL; SI: mmol/L, reference range: 2.1-2.6 mmol/L) and ionized calcium levels (mmol/L, reference range: 1.14-1.29 mmol/L; Sl: units/range are the same as conventional). Right vertical axis demonstrates discrete data for PTH levels (pg/mL; reference range: 10-65 pg/mL; SI: pmol/L, reference range 1.0-7.0 pmol/L). Ca, calcium; Hr, hour; iCal, ionized calcium; Post-op, postoperative; PTH, parathyroid hormone. * Postoperative day 3, at the time of reported hearing loss.

## Learning points

- Acute hypocalcemia from hungry bone syndrome after parathyroidectomy may present with atypical symptoms, including sensorineural hearing loss, in addition to classic neuromuscular findings.
- Calcium plays a critical role in cochlear hair cell function, and abrupt postoperative shifts in calcium homeostasis may impair auditory transduction.
- Early recognition of atypical symptoms of hypocalcemia, and treatment with aggressive calcium and calcitriol repletion, are essential to optimize outcomes.

## Contributors

All authors made individual contributions to the authorship. D.L. was involved in the diagnosis, surgery, and outpatient management of the patient. S.E., A.Y., and L.B. were involved in postoperative inpatient care. All authors participated in the writing and editing of the report and approved the final draft.

## Data Availability

Data sharing is not applicable to this article as no datasets were generated or analyzed during the current study.
